# The microscopic structure of charge density waves in underdoped YBa_2_Cu_3_O_6.54_ revealed by X-ray diffraction

**DOI:** 10.1038/ncomms10064

**Published:** 2015-12-09

**Authors:** E. M. Forgan, E. Blackburn, A. T. Holmes, A. K. R. Briffa, J. Chang, L. Bouchenoire, S. D. Brown, Ruixing Liang, D. Bonn, W. N. Hardy, N. B. Christensen, M. V. Zimmermann, M. Hücker, S. M. Hayden

**Affiliations:** 1 School of Physics & Astronomy, University of Birmingham, Birmingham B15 2TT, UK; 2 Physik-Institut, Universität Zürich, Winterthurerstrasse 190, CH-8057 Zürich, Switzerland; 3 XMaS, European Synchrotron Radiation Facility, B.P. 220, Grenoble F-38043, France; 4 Department of Physics, University of Liverpool, L69 3BX Liverpool, UK; 5 Department of Physics & Astronomy, University of British Columbia, Vancouver, British Columbia, Canada V6T 1Z1; 6 Department of Physics, Technical University of Denmark, DK-2800 Kongens Lyngby, Denmark; 7 Deutsches Elektronen-Synchrotron DESY, 22603 Hamburg, Germany; 8 Condensed Matter Physics & Materials Science Department, Brookhaven National Laboratory, Upton, New York 11973, USA; 9 H. H. Wills Physics Laboratory, University of Bristol, Bristol BS8 1TL, UK

## Abstract

Charge density wave (CDW) order appears throughout the underdoped high-temperature cuprate superconductors, but the underlying symmetry breaking and the origin of the CDW remain unclear. We use X-ray diffraction to determine the microscopic structure of the CDWs in an archetypical cuprate YBa_2_Cu_3_O_6.54_ at its superconducting transition temperature ∼60 K. We find that the CDWs in this material break the mirror symmetry of the CuO_2_ bilayers. The ionic displacements in the CDWs have two components, which are perpendicular and parallel to the CuO_2_ planes, and are out of phase with each other. The planar oxygen atoms have the largest displacements, perpendicular to the CuO_2_ planes. Our results allow many electronic properties of the underdoped cuprates to be understood. For instance, the CDWs will lead to local variations in the electronic structure, giving an explicit explanation of density-wave states with broken symmetry observed in scanning tunnelling microscopy and soft X-ray measurements.

## Abstract (web summary)

Near to the superconducting state, cuprates display spatially-periodic charge density variations. Here, the authors use x-ray diffraction to determine the microscopic structure, showing how charge density waves in underdoped YBa_2_Cu_3_O_6.54_ break the symmetry of the superconducting layers.

## Graphical abstract

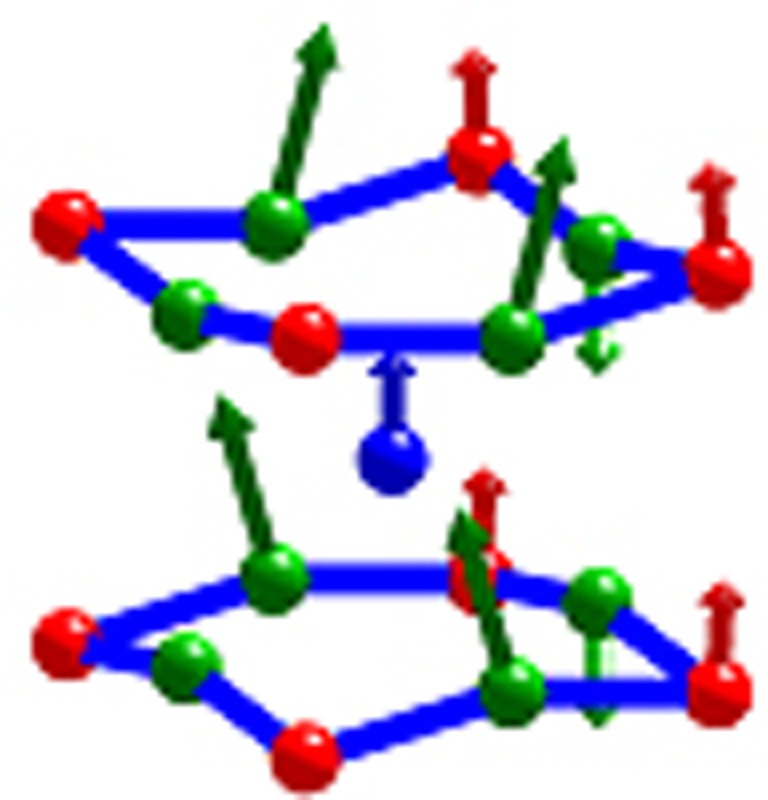

A charge density wave (CDW) is a periodic modulation of the electron density, associated with a periodic lattice distortion that may or may not be commensurate with the crystal lattice. The charge density modulation may be brought about by electron–phonon or electron–electron interactions^1^. It is now clear that the CDW state is a ubiquitous phenomenon in cuprate high-critical-temperature (high *T*_c_) superconductors, appearing in the underdoped region in both hole-2,3,4,5,6,7,8,9,10,11,12,13,14,15 and electron-doped^16^ materials at a temperature higher than *T*_c_, suggesting that the CDW is a characteristic instability of the CuO_2_ plane. The CDW competes with superconductivity^2^,^3^,^4^,^17^, and pressure-dependent data^18^ suggest that if the CDW can be suppressed in YBa_2_Cu_3_O_y_ (YBCO), then an enhanced *T*_*c*_ occurs in the nominally underdoped region rather than at optimum doping. Experiments on YBCO using resonant soft X-ray scattering suggest that the CDW is associated with significant *d*-wave components for charges on the oxygen bonds around the Cu site^19^,^20^, as proposed by Sachdev^21^. This conclusion is supported by scanning tunnelling microscopy (STM) observations of the surface of Bi_2_Sr_2_CaCu_2_O_8+x_ and Ca_2−x_Na_x_CuO_2_Cl_2_ (ref. 22). However, to understand the generic high-*T*_c_ CDW phenomenon, discovering the actual structure of the CDW is vital.

CDWs break the translation symmetry of the parent lattice, and have been observed by X-ray diffraction, and many other probes such as STM^22^ and nuclear magnetic resonance^17^,^23^. Signatures of the Fermi surface reconstruction believed to be associated with this include quantum oscillation measurements^24^, which show unexpectedly small Fermi surface pockets in an underdoped sample, and transport measurements, which indicate a change from hole carriers in the overdoped region to electron-like transport in the underdoped region^25^. To relate these observations to the CDW, its actual structure needs to be known. The studies of CDWs by X-ray diffraction in numerous cuprates have generally concentrated on determining the wave vector of the CDW and the temperature and magnetic field dependence of the order parameter and correlation lengths, and therefore have considered only a handful of diffraction satellites arising from the CDW. The only way to determine the structure unambiguously is by measuring the intensities of as many CDW satellites as possible.

Here we determine the structure of the CDWs in a bilayer cuprate. The material we have investigated is the well-studied material YBCO at a doping level where there is strong competition between superconductivity and the CDW, and the oxygen ordering in the crystal is most perfect. We find that the ionic displacements associated with the CDWs are maximum near the CuO_2_ bilayers and break their mirror symmetry. They involve displacements of planar oxygens perpendicular to the layers; these displacements have a strong component with *d*-symmetry. These results allow a physical understanding of the changes in electronic structure, transport properties and quantum oscillation results in the normal state of this cuprate material that are associated with the CDWs.

## Results

### X-ray diffraction measurements

We have used non-resonant X-ray diffraction to measure the intensities of all experimentally accessible CDW satellites near the (*h*, 0, ℓ), (0, *k*, ℓ) and (*h*, *h*, ℓ) planes for both of the CDW modulation vectors, **q**_a_=(**δ**_a_, 0, 0.5) and **q**_b_=(0, **δ**_b_, 0.5). Throughout this paper, we express wave vectors in reciprocal space coordinates (*h*, *k*, ℓ), where **Q**=(*h*a*+*k*b*+ℓc*). Here **a**, **b**, **c** are the Cartesian vectors defining the YBCO crystal cell dimensions, and a*, b*, c* are the corresponding vectors in reciprocal space; **q** is used to denote the full wave vector of a CDW mode and **δ** its basal plane part. By collecting a comprehensive data set, we deduce with great certainty the displacement patterns of the ions in the unit cell and hence the structure of the CDW in YBCO.

Our experiment was carried out on an underdoped crystal with the ortho-II structure (meaning that the oxygen sites on alternate CuO chains are unoccupied). The crystal was the same as that used in ref. 7. Ortho-II was selected as it has been well-studied by multiple techniques and the satellites associated with the oxygen ordering have minimal overlap with the CDW satellites. Here **δ**_a_∼0.323, **δ**_b_∼0.328 for this underdoped YBCO crystal.

Measurements were made at the superconducting *T*_c_ of our sample (60 K), where the CDW intensity is a maximum in zero field^4^. CDW signals in high-*T*_c_ cuprates are observed with basal plane wave vectors along both **a** and **b** crystal directions. These modulations may be present in the crystal in separate domains having **q**_a_ or **q**_b_ modulation (a 1-**q** model); alternatively, both modulations could be present and superposed in the same region (a 2-**q** model). Intensity measurements at separate **q**_a_ and **q**_b_ do not interfere, so all qualitative features of the two CDW components that we may deduce from our results are independent of the 1- or 2-**q** state of the sample, which only affects numerical estimates of the absolute magnitudes of the displacements (by a factor of √2).

**Figure 1:**
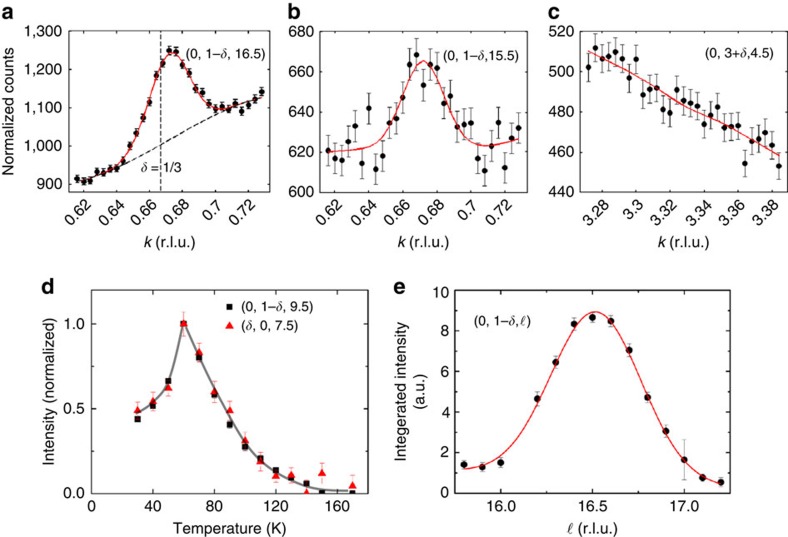
Typical observations of CDW satellites at 60 K and their temperature dependence. (**a**–**c**) are obtained for the CDW with modulation vector **q**_b_. They are scans parallel to the basal plane component of the modulation, through the points (*h*, *k*, ℓ)=(0, 1−**δ**, 16.5), (0, 1−**δ**, 15.5) and (0, 3+**δ**, 4.5). The counts are normalized so that they are approximately per second, measured over 10 s per point, plotted versus wave vector along the b* direction, labelled *k*. (**a**) shows a strong satellite, along with the fit line which gives the intensity as the area under the peak. The CDW is clearly centred at an incommensurate position (**δ**_b_∼0.328), although the value ⅓ lies within the peak. (**b**) shows a weaker peak and (**c**) is taken at a position where the CDW signal is unobservably small, and the fitted area of the peak is controlled by Poisson errors. (**d**) The intensities of CDW satellites for both **q**_a_ (**δ**_a_∼0.323) and **q**_b_ (**δ**_b_∼0.328) modes, normalized to their intensities at *T*_c_, are plotted versus temperature; these track each other within errors. (**e**) The integrated intensity of the satellite (**a**) is plotted versus ℓ. The width in ℓ, which reflects the finite *c*-axis coherence of the CDW, is much larger than the instrument resolution. Since it is a property of the CDW, it is the same for all satellites. All error bars in the above plots represent Poisson counting s.d.'s.

**Figure 2:**
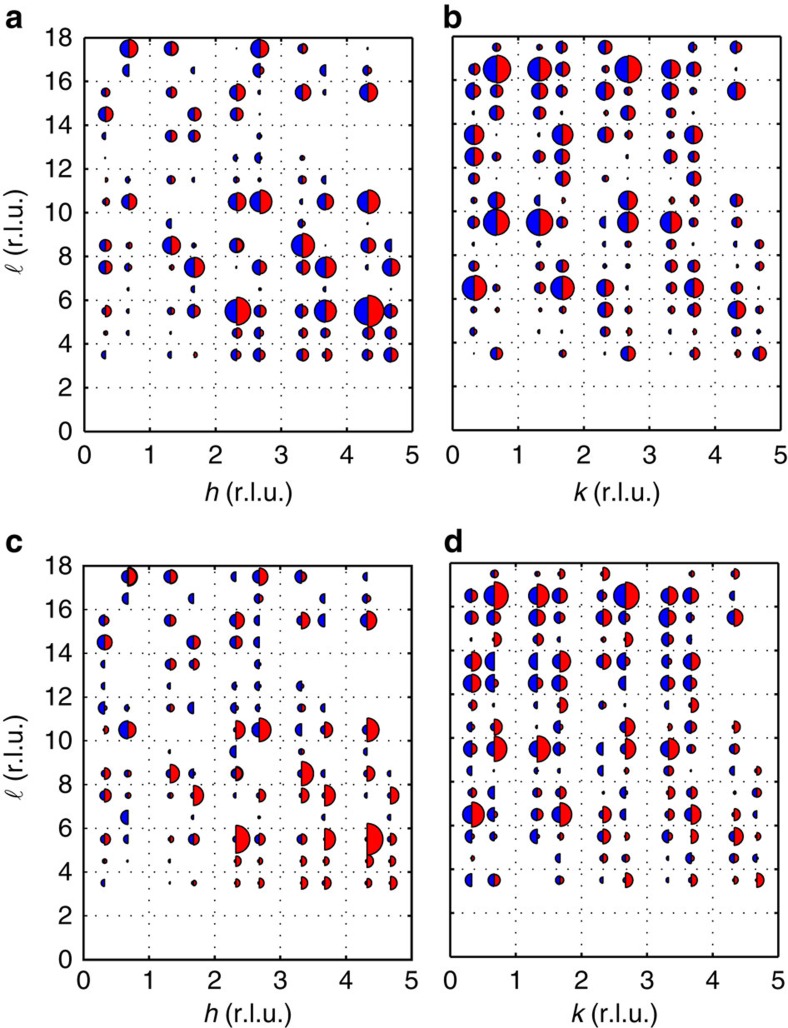
Sample data compared with the fits to the two possible models. (**a**,**b**) Maps of the satellite intensities associated with CDW modulations **q**_a_ and **q**_b_ measured in the (*h*, 0, ℓ) and (0, *k*, ℓ) planes of reciprocal space, respectively. The measured intensities are proportional to the areas of the red semicircles on the right of each **Q** point. Two different models are allowed by group theory: one involves the modes *A*_1_ (for **q**_a_) and *B*_1_ (for **q**_b_), and the other the *A*_3_ and *B*_3_ modes. The blue semicircles show the results of a good fit to the *A*_1_/*B*_1_ modes (to all measured data, not just that shown in the Figure). (**c**,**d**) The same data (red semicircles) and the analogous fits (blue semicircles) to the *A*_3_/*B*_3_ modes; these give a very poor fit to the data. Blank spaces indicate inaccessible regions or where a spurious signal prevented measurements of the CDW order.

Figure 1a–c shows some typical scans through CDW diffraction satellites. They peak at half-integral values of ℓ (Fig. 1e), and are extremely weak (∼10^−7^ of a typical crystal Bragg reflection). Therefore, the satellites are measured above a relatively large background, but due to their known position and shape, their intensities can be found and spurious signals ignored (see Methods). A compilation of some of the measured CDW intensities is displayed in Fig. 2; the area of the red semicircles is proportional to the measured peak intensity.

### X-rays are sensitive to ionic displacements

Non-resonant X-rays are primarily sensitive to the ionic displacements associated with a CDW, rather than changes in charge densities, although if one of these is present, so must the other^26^. (See Supplementary Note 1 for a simple model.) The CDW order gives rise to very weak diffraction satellites at positions in reciprocal space **Q**=**τ**±**q** around lattice Bragg peaks **τ** which are at integer *h*, *k* and ℓ. The diffracted amplitude at wave vector **Q** due to an ion carrying a total of *N* electrons displaced by small distance **u** is ∼*N* **Q.u**. Hence, the variation of the intensities with **Q** reflects the directions and magnitudes of the different ion displacements throughout the unit cell, and by observing intensities of CDW diffraction signals over a wide range of directions and values of **Q** we can determine the CDW structure. (See also an illustration of this point in Supplementary Fig. 1.) The full theory relating the CDW satellite intensities to the CDW structure is given in Supplementary Note 2.

We may write the displacements **u**_j_, of the individual ions from their regular positions 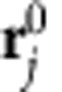 as a sum of two terms, one of which is polarized along **c** 
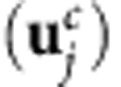
 and the other (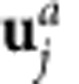 or 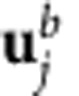) parallel to **δ**, with mirror symmetry about the relevant layer of the crystal.


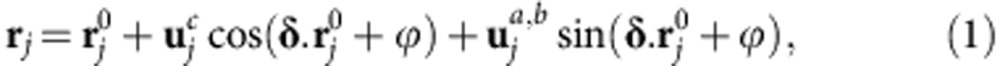


Symmetry^27^ requires that the 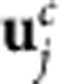 and 
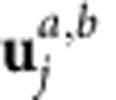
 displacements are π/2 out of phase, as expressed in equation (1).

### Ionic displacements obtained from the intensities

Group theory indicates which of the incommensurately modulated displacement patterns or irreducible representations (IRs) are consistent with the observed ordering wave vectors. There are four IRs for each ordering wave vector, labelled *A*_1_, *A*_2_, *A*_3_ and *A*_4_ for **q**_a_ and similarly *B*_1_–*B*_4_ for **q**_b_ (ref. 27). The even-numbered patterns have purely basal plane transverse displacements, and are therefore incompatible with our observations of satellites close to both the c* axis and the basal plane^4^. The other IRs have longitudinal displacements in the basal plane parallel to ***δ***, combined with shear displacements parallel to the *c* axis. Only the *A*_1_ pattern for **q**_a_ (and *B*_1_ for **q**_b_) are consistent with our data. These IRs have equal *c*-axis shear displacements in the two halves of the CuO_2_ bilayer region, combined with basal plane compressive displacements (and hence charge density modulations), which are equal and opposite in the two halves of a bilayer. Thus, these CDWs break the mirror symmetry of the bilayers. For these patterns, the CuO chain layer is a mirror plane of the CDW. For the patterns *A*_3_ and *B*_3_, the yttrium layer is instead a mirror plane of the CDW, so that the basal plane compressive displacements would be equal on the two sides of the bilayer. (See also Supplementary Note 3 and Supplementary Figs 2 and 3 for a visualization of these symmetries.)

Some ionic displacements in these IRs are zero by symmetry. This results in a detailed description of an IR that consists of 13 non-zero parameters representing displacement components of the 11 ions in the YBCO unit cell. In our model, we average over the half-occupied chain oxygen site in ortho-II YBCO, because we find no evidence for different responses in those cells having a full or empty CuO chain. (No CDW satellites were observed about **τ**+½a* positions). Our most complete data set is for the **q**_b_ satellites. The **q**_a_ data are somewhat sparser and the results have larger errors, due to the tails of the peaks arising from the ortho-II oxygen ordering which give large and rapidly varying backgrounds.

Models *A*_1_ and *B*_1_ always converged in a few iterations to a good fit and gave the same fitted values of displacements independent of the starting value of the parameters. In contrast, models *A*_3_ and *B*_3_ always gave poor fits (for example, *χ*^2^(*B*_3_) >10 × *χ*^2^(*B*_1_)), whatever the starting values of the fitting parameters. Sample data and fits are shown in Fig. 2, with complete maps of the intensities measured and the fits in Supplementary Fig. 4.

**Figure 3:**
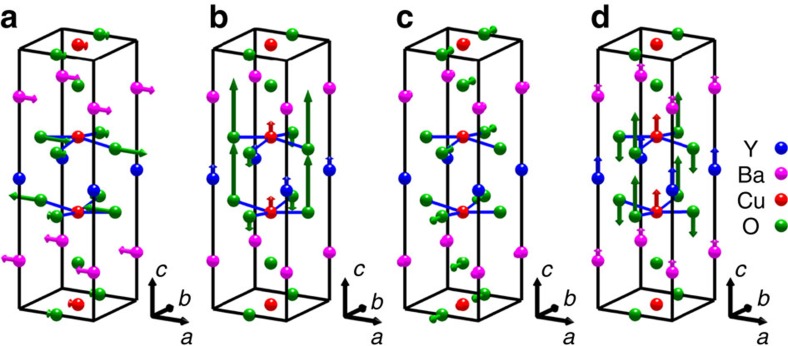
Representation of the CDW ionic displacement motifs for an unmodulated unit cell. The displacements are ∼10^−3^ of interatomic distances, so have been exaggerated to make them visible. In (**a**) and (**b**) are shown the a- and *c*-axis components of the **q**_*a*_ (*A*_1_) mode of the CDW. In (**c**) and (**d**) are shown the b- and *c*-axis components of the **q**_b_ (*B*_1_) mode. The basal plane and *c*-axis displacements have a *π*/2 phase difference and hence are shown in separate unit cells. The next crystal unit cells in the *c*-direction would be in antiphase with those shown here. These motifs are modulated as a function of position with the relevant wave vector. The oxygen sites in the CuO chains represented here are half-occupied in our sample.

**Figure 4:**
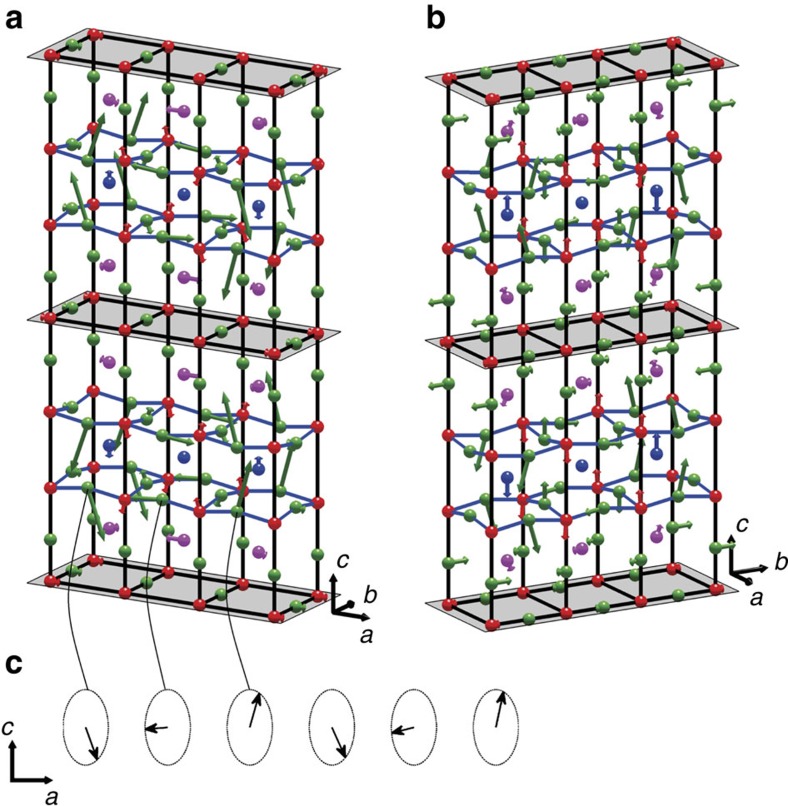
Representation of modulated ionic displacements for the CDW modes. The displacements are ∼10^−3^ of interatomic distances, so have been exaggerated to make them visible. In **a** is shown the spatial variation of the fitted ionic displacements for the *A*_1_ CDW mode with wave vector **q**_a_, and in (**b**) the fitted displacements for the *B*_1_ CDW mode with wave vector **q**_b_. The shaded planes passing through the CuO chain layers are the mirror planes of the CDWs. If the structure of the CDW is 1-**q**, these displacement patterns would be located in different regions of the crystal. If 2-**q**, the total displacement of the ions in the crystal would be the sum of those associated with the **q**_a_ and **q**_b_ modulation vectors. (**c**) The displacement of any particular ion in a CDW lies on an ellipse: we give an example for an oxygen in the lowest CuO_2_ plane of **a**.

We therefore conclude that the IRs *A*_1_ and *B*_1_ are close to an accurate representation of the CDW. In Fig. 3, we represent the patterns of ionic displacements in a single unit cell as given by the data for both **q**_a_ and **q**_b_ modulations. The overall similarity of the two patterns is apparent. The spatial variation of the ionic displacements, shown in Fig. 4, is derived from the motifs in Fig. 3 by modulating the *c*-axis and basal plane displacements by cos(2*πδ*_a_*x*/*a*) and sin(2*πδ*_a_*x*/*a*) respectively for the **q**_a_ mode, and similarly for **q**_b_.

We have obtained an estimate of the absolute magnitude of ion displacements by comparing the satellite intensities with those of the Bragg peaks from the lattice. (See Supplementary Note 4 for details.) The fitted values of the ionic displacements are given in Table 1. The table gives signs, values and errors for the fitted displacements from models *A*_1_ and *B*_1_ for the **q**_a_ and **q**_b_ modes. Certain features of our fit, such as the *c*-axis motion of the yttrium layer moving with both the CuO_2_ planes are expected on physical grounds, but were not imposed as constraints, giving extra confidence in the fit results. Most ionic displacement values are well-defined, but some oxygen horizontal displacements have large errors, particularly, that of the chain oxygen, which gives a small scattering amplitude, since it has a small number of electrons, is half-occupied and its amplitude falls off at large *Q*. The first pair of columns show the results for the **q**_b_ mode if the chain oxygen displacement is left free. It refines to a very large value with an even larger uncertainty. In the next pair of columns this displacement is set to a physically reasonable value equal to the small displacement of the adjacent Cu. It can be seen that this makes very little difference to the fitted values of the other ion displacements and to the *χ*^2^. (A small value of the chain oxygen displacement is justified on the grounds that soft X-ray measurements^3^ indicate very small CDW charge build-up in the chain layer.)

## Discussion

Earlier measurements found differences in the magnitudes of the X-ray signals from the **q**_a_ and **q**_b_ modes^6^,^7^ that suggested that the CDW in ortho-II YBCO might be essentially single-**q**, and dominated by the **q**_b_ mode. The results presented here indicate that this is not the case; the two modes have similar displacement amplitudes, but the value of their ratio depends on which ion is chosen to make the comparison. For instance, if we consider the motion of the CuO_2_ plane oxygens as key, we find that the relative *c*-motion of these oxygen ions is essentially identical for the two modes: in both cases, the amplitude is ∼4–5 × 10^−3^ Å. Our fits do show differences in the heavy ion displacements, and even if these are small, they can make noticeable contributions to the X-ray signals because these ions carry many core electrons. Only this complete survey, rather than measurements of the intensities of a few **q**_a_ and **q**_b_ satellites, can reveal the similarities and differences between the two CDW modes.

The deduced ionic displacements are maximal near the CuO_2_ planes and weak near the CuO chains; this is in agreement with the observed competition between the CDW and superconductivity^2^,^3^,^4^,^17^. Surprisingly, the largest amplitudes are out-of-plane shear rather than compression of the CuO_2_ planes, so that the CDW is not purely a separation of charge as commonly assumed. It may be that the lattice is deforming in this way because shear deformations cost less elastic energy than compressive ones. There are also CDW-modulated charges associated with the small longitudinal displacements in the two halves of a bilayer, but they are equal and opposite. This would be favoured by Coulomb effects within a bilayer. We note the similarity of some of the displacements to a soft phonon observed in optimally doped YBCO (ref. 28). However, in that mode, the *c*-motion is in antiphase for the two halves of the bilayer. Buckling of the CuO_2_ planes is also seen in 214 compounds^29^, where it mainly consists of tilts of rigid Cu–O octahedra. Here, however, the displacements in the CuO_2_ layers are clearly inconsistent with tilts of a rigid arrangement of ions.

**Figure 5:**
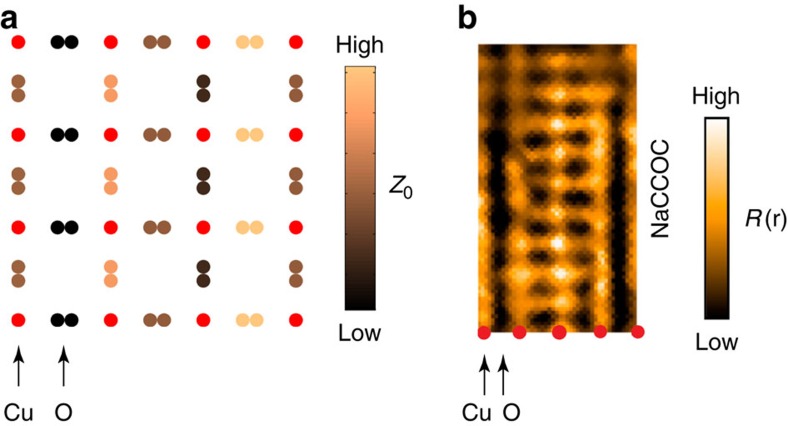
Comparison of the bilayer oxygen height with an STM R(r) image. (**a**) A representation of the spatial variation of the *z* co-ordinate of the bilayer oxygen atoms from our X-ray results is shown for one (**q**_a_) of two modulation directions present in the crystal. In (**b**) is an STM *R*(**r**) image, (where *R*(**r**)=*I*(**r**,*E*)/−*I*(**r**,−*E*) is a measure of the asymmetry of the current for positive and negative bias) acquired from the lightly doped cuprate Ca_2-x_Na_x_CuO_2_Cl_2_ (NaCCOC). (Reproduced with permission from ref. 22.) The *R*(**r**)-image is used to highlight spatial variation of doping or electronic structure. Both **a** and **b** have the same symmetry as a bond *d*-density wave along the **δ**_a_ direction. Note that NaCCOC has a repeat period of approximately four unit cells, which is longer than that in YBCO.

We draw attention to the up/down butterfly nature of the displacements of the four oxygens around a Cu in the bilayers, which is seen for both **q**_a_ and **q**_b_ modes. The two oxygens in the **δ**-direction around a copper are displaced in the same direction as the Cu along **c**, but the other perpendicular pair is displaced oppositely (Fig. 3). To an STM (ref. 22) this could appear as a *d*-charge density on the oxygens, since *c*-axis motion of an oxygen—relative to the yttrium and/or to the crystal surface would alter its local doping and electronic state. We note that the STM measurements are analysed in such a way as to emphasize the electronic states, rather than the positions of atoms. In Fig. 5a, we show qualitatively what the effect on the local doping of the oxygen ions might be by assuming that the change is proportional the displacement along **c**. The pattern produced has the same symmetry as that observed by STM (ref. 22) in Ca_2−*x*_Na_*x*_CuO_2_Cl_2_ (Fig. 5b). STM and azimuthal angle-dependent resonant X-ray studies^19^,^20^ of the charge order have been analysed in terms of modulated states with local symmetry of three types with respect to a planar copper site: equal density on the copper atoms (*s*-symmetry); equal density on the neighbouring oxygen atoms (*s*′-symmetry); opposite-sign density on the neighbouring O_*x*_ and O_*y*_ sites (*d*-symmetry). Our measured copper and oxygen displacements, recorded in Table 1, can provide an explanation for the relative proportions of these components. In agreement with the STM and resonant X-ray studies, we find that the *d*-symmetry component is dominant.

These results carry several important messages. First, they show that a strictly planar account of high-*T*_c_ phenomena may miss important aspects of the physics, and that the third dimension and crystal lattice effects cannot be ignored. In our experiments, we have observed a charge density wave with a strong shear (*c*-axis) component. The butterfly pattern of oxygen shear displacements around the planar copper ions can simulate a *d*-charge density on the oxygens. It will be very interesting to repeat these X-ray measurements on other underdoped high-*T*_c_ compounds to establish the generality (or otherwise) of these results, and to relate these results to the changes in the CDW that occur at high fields where quantum oscillation measurements are performed. Ultrasonic measurements^30^ show that changes occur at ∼18 T. Very recent measurements in pulsed field^31^ in an YBCO sample with ortho-VIII oxygen ordering show that longer-range order with the same value of **δ**_b_ emerges at high field. This is clearly related to our zero-field structure, and leads to interesting questions^32^ about the Fermi surface reconstruction at low and high fields. It is clear that antiferromagnetic order, the CDW, pseudogap and superconductivity are all intertwined, since they all remove electron states near the antinodal regions of the Fermi surface. It appears that there is a quantum critical point underlying the superconducting dome; we trust that our results will help to achieve an overarching theory relating the relationship of all these phenomena to high-*T*_c_ superconductivity.

## Methods

### X-ray techniques

To obtain sufficient data required the flexibility of a four-circle diffractometer, which is provided at the XMaS beamline (XMaS—The UK material science beamline at the ESRF (2015): http://www.xmas.ac.uk) at the ESRF, Grenoble^33^. The sample was mounted in a closed-cycle cryostat and all measurements were carried out in zero magnetic field in reflection from the flat *c* face of the crystal (of area ∼2 × 2 mm^2^) at an X-ray energy of 14 keV. This gives a penetration depth of 25 μm into the sample, so the results are not dominated by surface effects. For CDW intensity measurements, the sample temperature was controlled at *T*_c_ to maximize the signal, and it was taken to 150 K to check for spurious signals, which did not go to zero. The diffractometer angles were set so that the incoming and detected beams were close to the same angle to the *c* face of the crystal, which allowed correction for sample absorption, as described in Supplementary Note 5. CDW intensity measurements were carried out near the (*h*, 0, ℓ), (0, *k*, ℓ) and (*h*, *h*, ℓ) planes of reciprocal space over as wide a range of *h*, *k* and ℓ allowed by the maximum scattering angle, and the avoidance of grazing incidence at low ℓ. CDW peaks were scanned parallel to **δ**, through positions of the form **Q**=**τ**±**q**.

### Measurement and fitting of CDW satellite intensities

The intensities of the CDW peaks were established by fitting each scan with a Gaussian of fixed width, with a smoothly varying cubic polynomial background. By examination of 150 K measurements, or by the *χ*^2^ of the fit, spurious peaks were removed from the list of measured satellites. As shown in Fig. 2, the finite range of the CDW order results in satellites that are broad, particularly in the c* direction. However, all the intensity of any satellite is confined to a single Brillouin zone, allowing it to be integrated over reciprocal space. The resulting list of intensities, weighted by their errors, was fitted to our CDW models by varying the ionic displacements {**u**_*j*_} to minimize *χ*^2^. Further details are in Supplementary Note 2.

## Additional information

**How to cite this article:** Forgan, E. M. *et al*. The microscopic structure of charge density waves in underdoped YBa_2_Cu_3_O_6.54_ revealed by X-ray diffraction. *Nat. Commun.* 6:10064 doi: 10.1038/ncomms10064 (2015).

## Supplementary Material

Supplementary Information Supplementary Figures 1-4, Supplementary Notes 1-5 and Supplementary References.

## Figures and Tables

**Table 1:** The values of fitted ionic displacements.

	*u*_c_ for q_b_	*u*_b_ for q_b_	*u*_c_ for q_b_	*u*_b_ for q_b_	*f*(*Q*=0) × *u*_c_ (q_*b*_)	*f*(*Q*=0) × *u*_b_ (q_*b*_)	*u*_c_ for q_a_	*u*_a_ for q_a_	*f*(*Q*=0) × *u*_c_ (q_a_)	*f*(*Q*=0) × *u*_a_ (q_a_)
Y	1.50 (3)	0	1.50 (3)	0	54 (1)	0	0.94 (6)	0	34 (2)	0
Ba	0.83 (2)	0.66 (3)	0.83 (2)	0.65 (2)	45 (1)	35 (1)	−0.20 (2)	1.30 (5)	−11 (1)	70 (3)
Cu (plane)	1.49 (3)	0.17 (5)	1.48 (3)	0.18 (5)	40 (1)	5 (1)	1.06 (5)	0.42 (6)	29 (2)	11 (2)
O_*x*_ (plane)	−1.68 (16)	0.15 (30)	−1.66 (15)	0.0 (0)	−17 (2)	0 (0)	3.83 (30)	2.30 (42)	38 (3)	23 (4)
O_*y*_ (plane)	2.65 (16)	1.34 (30)	2.64 (16)	1.38 (27)	27 (2)	14 (3)	−0.94 (28)	0.67 (40)	−9 (3)	7 (4)
O (apical)	−0.08 (18)	1.46 (24)	0.0 (0)	1.44 (23)	0 (0)	51 (9)	0.0 (0)	0.0 (0)	0 (0)	0 (0)
Cu (chain)	0	0.58 (7)	0	0.58 (7)	0	16 (2)	0	0.71 (9)	0	19 (3)
O (chain)	0	1.4 (2.7)	0	0.58 (0)	0	3 (0)	0	0.71 (0)	0	4 (0)
D-W *α*	4.9 (5)	4.9 (5)	4.8 (5)	4.8 (5)			6.2 (9)	6.2 (9)		
D-W *β*	3.3 (10)	3.3 (10)	3.2 (9)	3.2 (9)			4.8 (16)	4.8 (16)		
*χ*^2^	1.01	1.01	1.00	1.00			0.96	0.96		

These are in absolute units, 10^−3^ Å, calculated, as described in Supplementary Note 4 and 5, from the fit of the data for the **q**_b_ and **q**_a_ modulated CDWs in ortho-II YBCO and subject to an overall possible systematic error ∼50%, not included above. In the first two columns all variables are free, and in the second pair some values have been fixed and are marked by an error 0 in parentheses. Also given are the values multiplied by the scattering amplitude for each ion at *Q*=0 (the number of electrons on the ion) to emphasize the relative contributions of each ion to the amplitude. The *c*-components of the displacements are even about the yttrium layer of the crystal unit cell, and the horizontal displacements are odd. Displacements that are zero by symmetry are represented by 0. Below the displacements are given the fitted anisotropic Debye–Waller factors *α* and *β*, which appear in the expression: 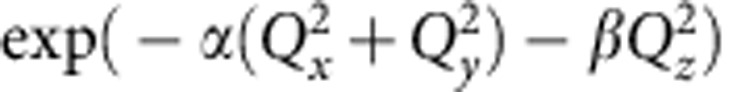. This multiplies the calculated intensities, and slightly improves the fit to the data, although the fitted displacements are little altered by including it. The units of *α* and *β* are 10^−3^ Å^2^. The bottom row of the Table gives *χ*^2^ per degree of freedom for the fits.
