# Supplementary material for: The microscopic structure of charge density waves in underdoped YBa2Cu3O6.54 revealed by X-ray diffraction
Source: Nat Commun. 2015 Dec 9;6:10064. doi: 10.1038/ncomms10064 (PMC4682044; doi:10.1038/ncomms10064)
Supplement: Supplementary Information — Supplementary Figures 1-4, Supplementary Notes 1-5 and Supplementary References. [file ncomms10064-s1.pdf]

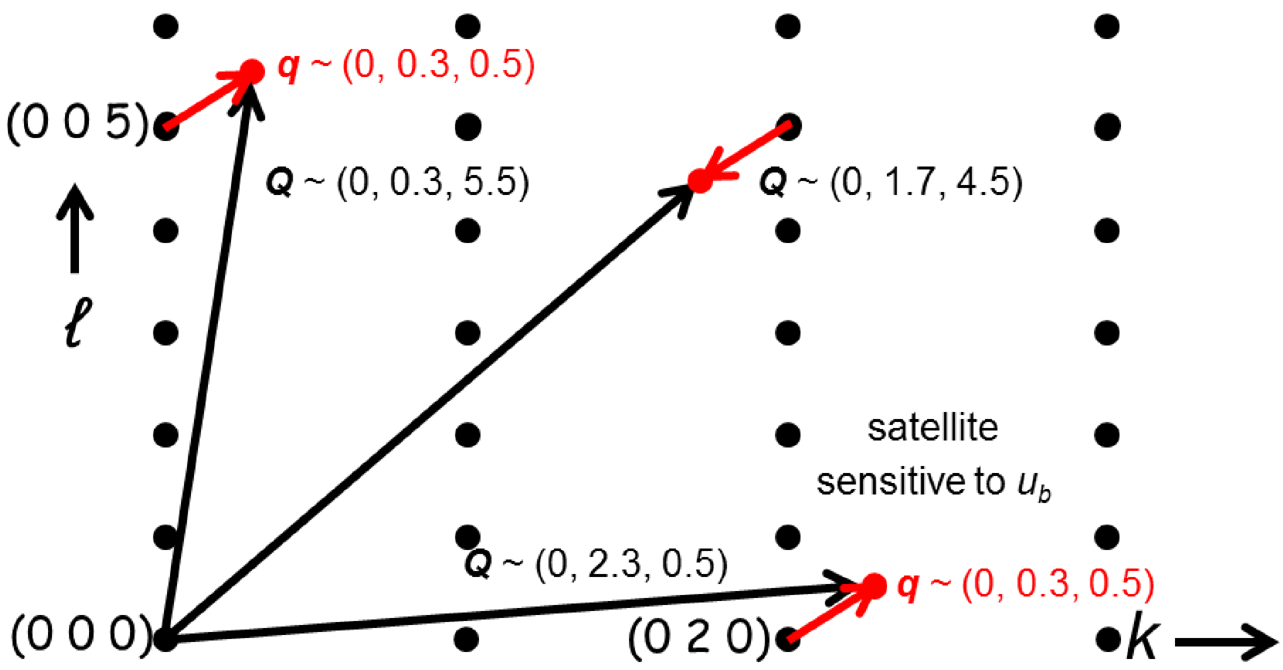

Supplementary Figure 1: Q-vectors for typical CDW satellites illustrating the Q.u effect

This shows an area of the **b*****c*** plane of reciprocal space, with lattice Bragg peaks marked in black, and some typical CDW satellites marked in red. Intensities of CDW satellites close to the horizontal **b*** axis are derived mainly from the sum of amplitudes for ionic displacements that are parallel to that axis. For satellites close to the **c*** axis, the vertical displacements will dominate. Well away from these axes, both directions of displacement interfere to give the total intensity.

(a)

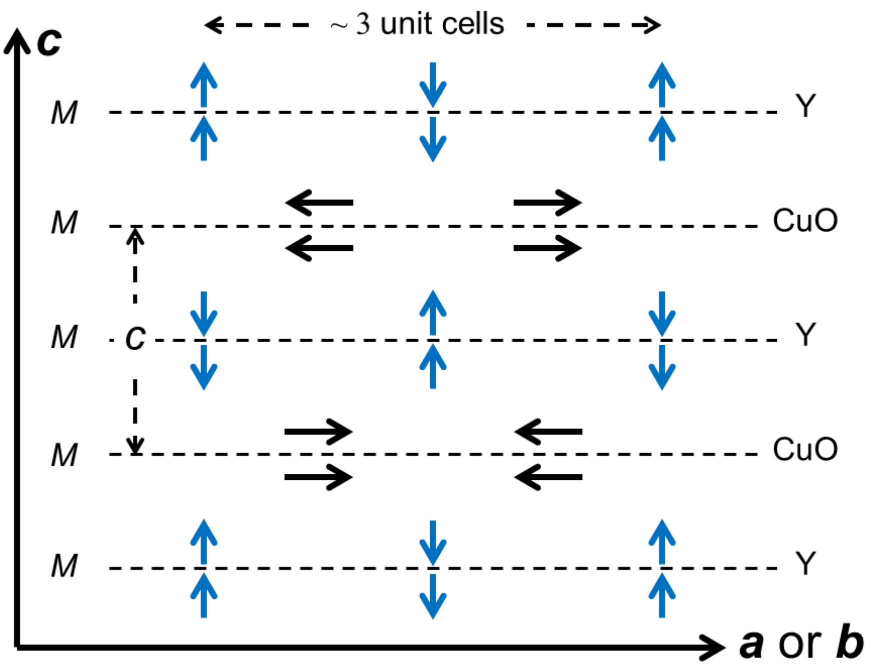

(b)

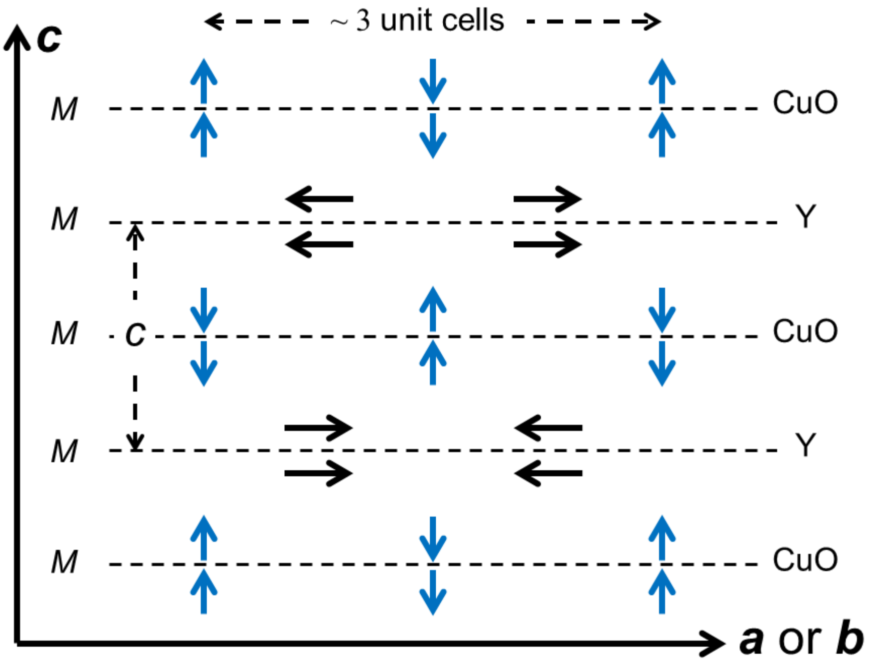

Supplementary Figure 2: Schematic indication of the possible symmetries of an incommensurate longitudinal CDW with ℓ = ½

The two mirror planes of the YBCO crystal structure perpendicular to the *c*-axis are marked *M* and labelled with Y or CuO, denoting respectively the centres of the bilayers or the CuO chains through which they pass. The symmetry of the displacement patterns for models A_1 and B_1 is shown in (a) and for

A_3 and B_3 in (b). The **c**-axis displacements have a phase difference of $\pi/2$ from the basal plane displacements but will be in phase with any charge density modulations associated with the basal plane displacements. The data clearly indicate that models A_1 and B_1 give the correct displacement patterns for modulations along the **a** and **b** directions respectively.

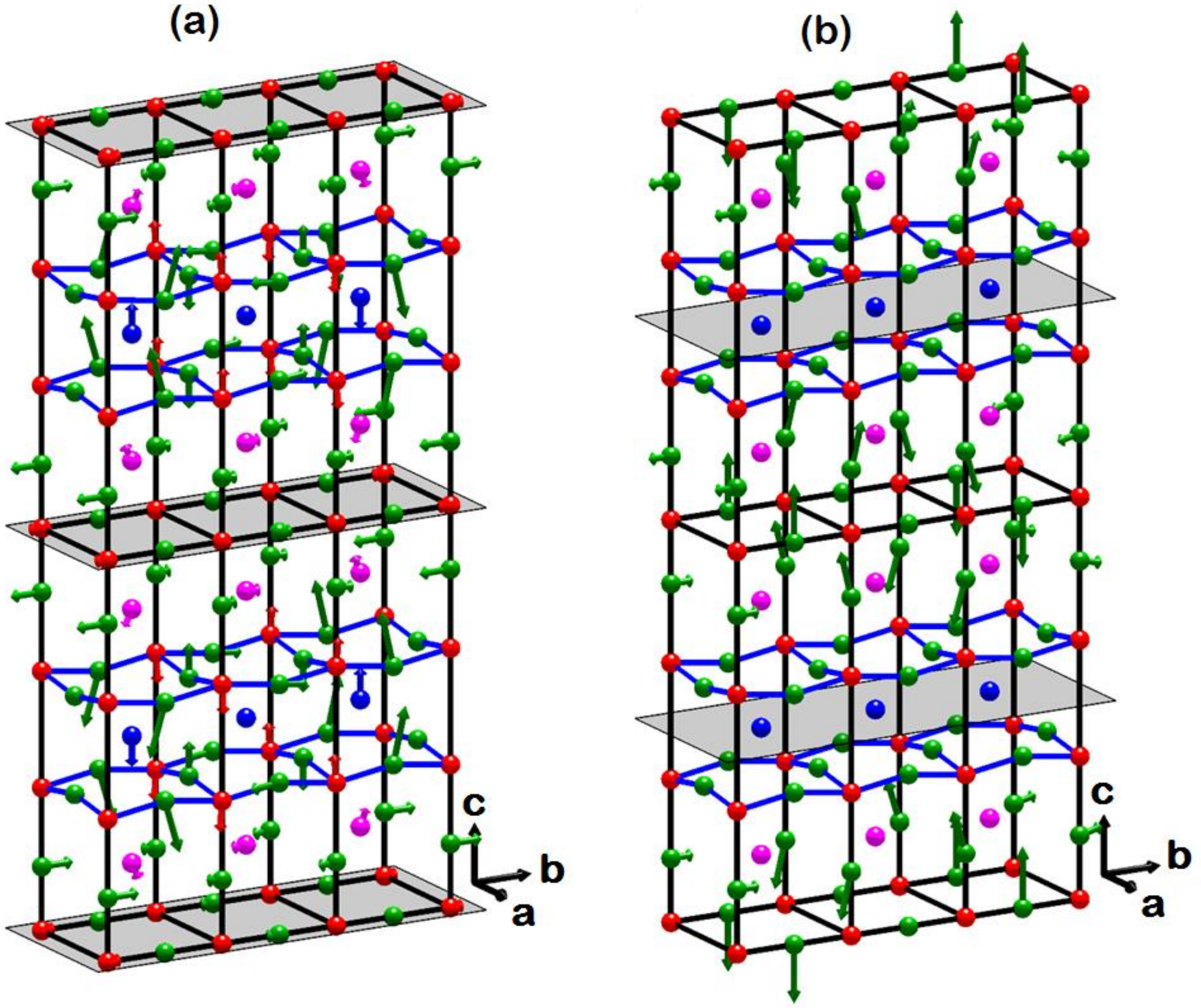

Supplementary Figure 3: Best fit of the B_1 pattern (IR) and 'least bad fit' of the B_3 pattern

In (a) is represented the pattern of displacements given by the model B_1 fit to the CDW with wavevector $\mathbf{q}_b$, and in (b) is shown the pattern from the model B_3 fit. Grey planes are the mirror planes of the two possible CDW structures. The χ^2 of the B_3 fit is an order of magnitude worse than for B_1. The B_3 displacements in (b) have been scaled down 6 times relative to the B_1 plot in (a), because the B_3 fit gives unphysically large displacements in the chain plane, as well as giving a very poor fit.

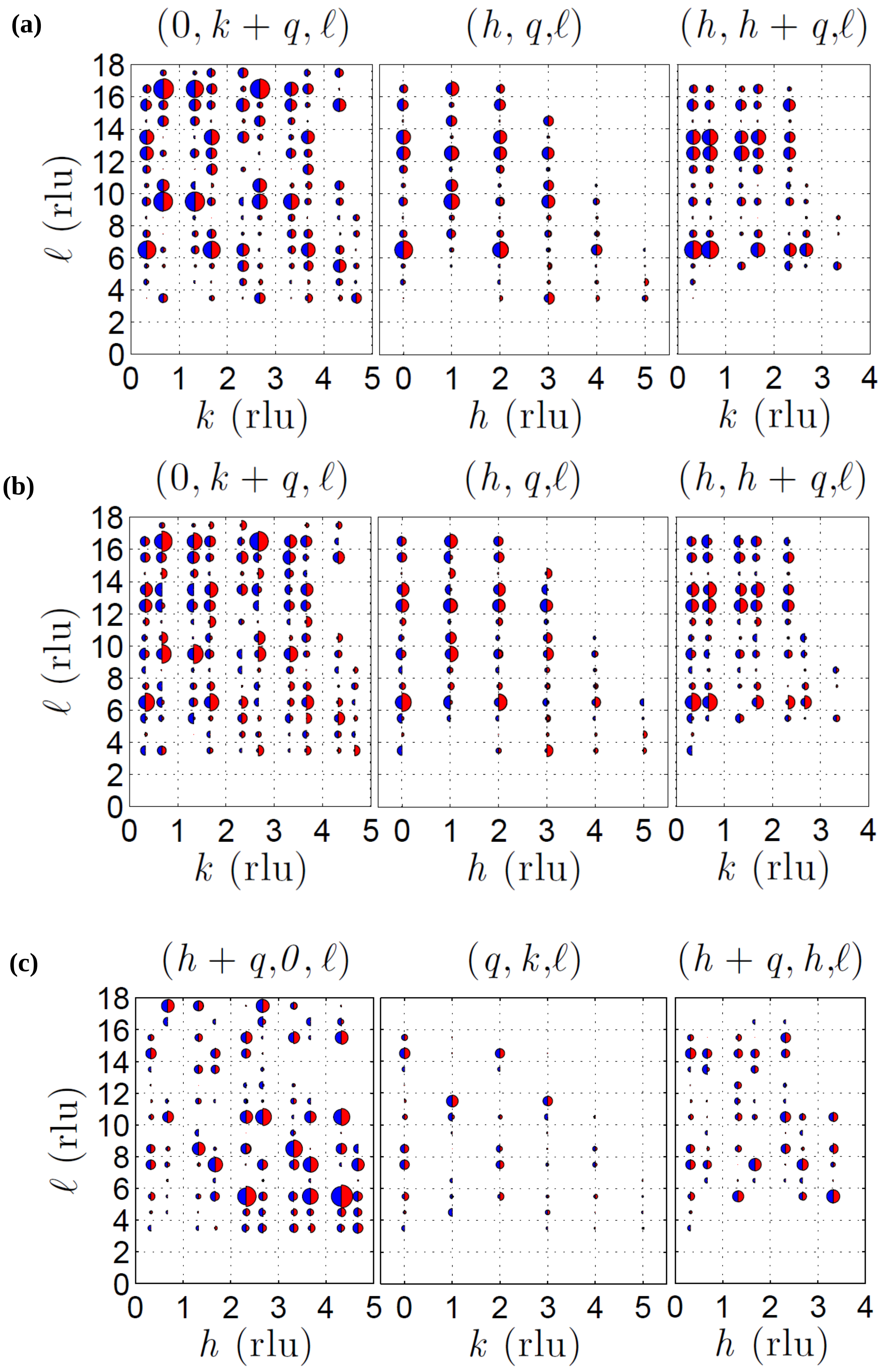
(a)
$(0, k+q, \ell)$
(h, q, ℓ)
$(h, h+q, \ell)$
ℓ (rlu)
k (rlu)
h (rlu)
k (rlu)
(b)
$(0, k+q, \ell)$
(h, q, ℓ)
$(h, h+q, \ell)$
ℓ (rlu)
k (rlu)
h (rlu)
k (rlu)
(c)
$(h+q, 0, \ell)$
(q, k, ℓ)
$(h+q, h, \ell)$
ℓ (rlu)
h (rlu)
k (rlu)
h (rlu)

Supplementary Figure 4: Maps of the CDW intensity data and their fits

These maps show the intensity data input to the fitting program and the patterns of intensity resulting from the fits to 272 intensity measurements for the $\mathbf{q}_b$ mode and 193 for $\mathbf{q}_a$. We plot the data (areas of red semicircles on the right at each point are $\propto$ intensity) for all three planes of reciprocal space that were measured, together with the fitted intensities (blue semicircles on the left). (a) the good fit to model B_1 for the $\mathbf{q}_b$ mode (b) the "least bad fit" from model B_3 for the same mode, which was inconsistent with the data (c) Good fit only – model A_1 - for the $\mathbf{q}_a$ mode. Blank regions of the maps indicate where data were not taken.

Supplementary Note 1: Contributions of displacements and charges to X-ray signals

This matter is discussed in Refs. 1 and 2 and we give a simplified discussion, appropriate to YBCO here. Consider a simple model where the displacement of an O^{2-} ion is associated with adjacent CDW charges of $\pm\delta q$ at distances $\pm d/2$, where d is the lattice spacing. The charges will give rise to an electric field and hence a force F on the ion given by:

$$|F| = 2eE = \frac{2e.2\delta q}{4\pi\varepsilon_0 (d/2)^2} \quad (1)$$

If the force constant holding the ion in place is k, then it will move to an equilibrium distance δr away from its previous position, with $\delta r = F/k$. An estimate of k may be obtained using $k \sim m\omega^2$, where m is the mass of the oxygen ion and ω the angular frequency of optical phonons in YBCO involving oxygen. The contribution of all the electrons on the displaced oxygen to the amplitude of the CDW structure factor at $\mathbf{Q}$ is $\mathbf{Q}.\delta\mathbf{r} \times 10e$. The smallest value of $|\mathbf{Q}|$ is $\sim 2\pi/d$. Combining these together, taking 0.5 as a typical value of the dot product, we find that the minimum ratio between the displacement contribution and the $2\delta q$ charge contribution to the CDW diffraction amplitude is

$$\frac{20e^2}{\varepsilon_0 d^3 m\omega^2} \quad (2)$$

We take $d \sim 3.8$ Å, and in YBCO, a typical value for ω is $2\pi \times 13$ THz. Inserting all values into the ratio gives a minimum value of the ratio ~6 for the amplitude, hence 36 for the intensity. We note that for shear motions, which are the main ones observed in YBCO, the CDW charge density modulations and force constants will be smaller, both of which increase the ratio. Also, any CDW charge densities will involve outer electrons, which will have form factors that fall off more rapidly with Q than the ionic form factors. Hence, the contribution of CDW charge modulations to the X-ray diffraction pattern may have a small effect at small Q for oxygen ions; at larger Q and for the heavier atoms the effects of ionic displacements will certainly be dominant.

Supplementary Note 2: Theoretical calculation of satellite intensities

The amplitude S of the X-ray structure factor at wavevector $\mathbf{Q}$ for a crystal may be written:

$$S(\mathbf{Q}) = \langle \sum_j f_j(Q) e^{i\mathbf{Q}.(\mathbf{R}_k+\mathbf{r}_j)} \rangle, \quad (3)$$

where the average is over unit cells centred at points $\mathbf{R}_k$ and over ions at points $\mathbf{r}_j$ within unit cells. The values at 60 K of the non-symmetry-determined values of the ionic positions $\boldsymbol{r}_j$ were refined from a fit to the lattice intensities, giving $z_{\mathrm{Ba}} = 0.3111$, $z_{\mathrm{Cu2}} = 0.1425$, $z_{\mathrm{O2}} = 0.1221$ and $z_{\mathrm{O4}} = 0.3442$ (all relative to Y, taken to have $z_{\mathrm{Y}} = 0$); these are close to the room temperature values for $O_{6.55}$ composition[3], but gave a better fit to both CDW and lattice intensities. The Q-dependent scattering amplitudes or 'form factors' f_j of each ion were taken from standard tables[4], using charges ±2 for Cu, Ba and O and +3 for Y; f for the O^{2-} ion was taken from Ref. 5. Small corrections to f_j for anomalous scattering at 14 keV were made[6].

The YBCO crystal structure is centrosymmetric about the Y position, and for simplicity of calculation we take the co-ordinate of Y as (0, 0, 0) with the unit cell extending ± $c/2$ in z. We re-express the patterns of displacements for the IRs given in equation (1) in the main text, in terms of their even or odd symmetry about the Y layer.

In a CDW, each ion is displaced from its regular crystal position $\mathbf{r}_j^0$ by small amounts $\mathbf{u}$:

$$\mathbf{r}_j = \mathbf{r}_j^0 + \mathbf{u}_j^e \cos(\boldsymbol{\delta}.\mathbf{r}_j^0 + \varphi) + \mathbf{u}_j^o \sin(\boldsymbol{\delta}.\mathbf{r}_j^0 + \varphi), \quad (4)$$

where $\boldsymbol{\delta}$ is the basal-plane part of the modulation vector, and we have labelled the displacements as those $\mathbf{u}_j^e$,which are even in z about the Y-layer and those $\mathbf{u}_j^o$,which are odd. IRs A_3 and B_3 have all the $\mathbf{u}_j^e$ parallel (or antiparallel) to $\boldsymbol{\delta}$ (e.g. along the $\mathbf{b}$-direction for δ_b) with the $\mathbf{u}_j^o$ along the ±$\mathbf{c}$-direction. IRs A_1 and B_1 (which fit the results) have the $\mathbf{u}_j^e$ along ±$\mathbf{c}$ and the $\mathbf{u}_j^o$ along ±$\boldsymbol{\delta}$. For an incommensurate modulation, the even and odd displacements necessarily have a phase difference of $\pi/2$, and the phase φ has no importance.

The appearance of CDW satellites around (ℓ+½) (ℓ integral) implies that the next unit cell at $+c$ in the z-direction has:

$$\boldsymbol{r}_j = \boldsymbol{r}_j^0 - \boldsymbol{u}_j^e \cos(\boldsymbol{\delta}.\boldsymbol{r}_j^0 + \varphi) - \boldsymbol{u}_j^o \sin(\boldsymbol{\delta}.\boldsymbol{r}_j^0 + \varphi) \quad (5)$$

The symmetries of the system result in $\mathbf{u}_{\mathrm{Y}}^o = 0$ and also $\mathbf{u}_{\mathrm{Cu}}^e = \mathbf{u}_{\mathrm{O}}^e = 0$ in the chain layer, resulting in 13 independent **u**-parameters in either model.

For small **Q.u** one may write:

$$\exp\left(i\mathbf{Q}.\left(\mathbf{r} + \mathbf{u}e^{\mp i\boldsymbol{\delta}.\mathbf{r}}\right)\right) = \exp(i\mathbf{Q}.\mathbf{r}) + i\mathbf{Q}.\mathbf{u}e^{i(\mathbf{Q}\mp\boldsymbol{\delta}).\mathbf{r}}. \quad (6)$$

The first term on the RHS corresponds to the lattice, and the average over unit cells gives Bragg peaks at reciprocal lattice points, i.e. $\mathbf{Q} = \boldsymbol{\tau} = h\mathbf{a}^* + k\mathbf{b}^* + \ell\mathbf{c}^*$. The second gives the CDW peaks at $\mathbf{Q} \mp \boldsymbol{\delta} = h\mathbf{a}^* + k\mathbf{b}^* + (\ell + ½)\mathbf{c}^*$, which we write as $\mathbf{Q} = \boldsymbol{\tau}' \pm \boldsymbol{\delta}$. We see that Supplementary Equation (6) encapsulates the sensitivity of the results to the directions of the ionic motions, as illustrated in Supplementary Figure 1.

Inserting Supplementary Equation (4) into (3) and averaging over unit cells, we obtain for the amplitude of the CDW structure factor:

$$S(\mathbf{Q} = \boldsymbol{\tau}' \pm \boldsymbol{\delta}) \propto \sum_j{}' \begin{Bmatrix} +i\mathbf{Q}.\mathbf{u}_j^e n_j f_j(Q)\left(e^{2\pi i\left(hx_j+ky_j+(\ell+½)z_j\right)} + e^{-2\pi i\left(hx_j+ky_j+(\ell+½)z_j\right)}\right)e^{\mp i\varphi} \\ \mp\mathbf{Q}.\mathbf{u}_j^o n_j f_j(Q)\left(e^{2\pi i\left(hx_j+ky_j+(\ell+½)z_j\right)} - e^{-2\pi i\left(hx_j+ky_j+(\ell+½)z_j\right)}\right)e^{\mp i\varphi} \end{Bmatrix} \quad (7)$$

The coordinates of each ion (x_j, y_j, z_j), relative to the yttrium taken as (0, 0, 0), are in units of the crystal lattice parameters a, b, and c. To obtain this expression, we have used the centrosymmetry of the unit cell, the sum is over the half-unit cell with $z \geq 0$ and n_j is the number of ions of type j in the cell (n_j = 1 for Y and chain Cu; n_j = 2 for Cu and both O_x and O_y in the CuO_2 layers and for Ba and O in the BaO layers; n_j = ½ for the O in the half-occupied chains.)

Finally we may write:

$$S(\mathbf{Q} = \boldsymbol{\tau}' \pm \boldsymbol{\delta}) \propto \sum_j{}' \begin{Bmatrix} +i\mathbf{Q}.\mathbf{u}_j^e n_j f_j(Q)\cos(2\pi\left(hx_j + ky_j + (\ell + ½)z_j\right))e^{\mp i\varphi} \\ \mp i\mathbf{Q}.\mathbf{u}_j^o n_j f_j(Q)\sin(2\pi\left(hx_j + ky_j + (\ell + ½)z_j\right)e^{\mp i\varphi} \end{Bmatrix} \quad (8)$$

The intensity of the CDW satellite depends on the square modulus of S, and we note that the phase factor φ disappears. The experimental results are fitted to this quantity, which we multiply by an overall Debye-Waller (D-W) factor that allows for any short-range disorder, and also corrects for any systematic dependence of the measured intensities on the diffractometer angles:

$$I(\mathbf{Q} = \boldsymbol{\tau}' \pm \boldsymbol{\delta}) \propto |S(\mathbf{Q} = \boldsymbol{\tau}' \pm \boldsymbol{\delta})|^2 \times exp\left(-\alpha(Q_x^2 + Q_y^2) - \beta Q_z^2\right) \qquad (9)$$

None of the qualitative features of the fits depend on the inclusion of a D-W factor, but it improves the χ^2 by some 20% and makes minor changes to magnitudes of $\mathbf{u}_j$. The calculations leading to Supplementary Equation (9) assume an ideal long-range-ordered CDW, which would lead to δ-function scattering at allowed values of $\mathbf{Q}$. In practice the CDW is short-ranged, which spreads out the δ-function into an ellipsoidal region containing the intensity. Under the conditions of our experiment, the dimensions of the ellipsoid were a property of the CDW and not the diffractometer setup, so were independent of $\mathbf{Q}$, and the relative intensities of different satellites could be obtained from a single scan (along $\boldsymbol{\delta}$) through the ellipsoid.

We note that the coherence length of the CDW is not long enough for the incommensuracy to sample all phases relative to the lattice in a single coherent region (a coherence length of ~ 20 unit cells gives a phase slip of ~ 0.4 π for (δ_a -⅓) ~ 0.01, and ~ 0.2 π in for δ_b). The CDW may have a lower energy for a particular phase and hence might accommodate slight incommensuracy (driven by band structure effects) by incorporating defects in the CDW. We cannot obtain the CDW phase relative to the lattice from intensity results – in appropriate cases this might be done by STM at the surface[7]. If we suppose that the CDW were locally commensurate, with period 3, then the phase factor ϕ would have a physical significance, and the ionic motions would only be approximately represented by Supplementary Equation (2) since each of the 3 unit cells representing the repeat period would be unique (implementing this in fitting would lead to an unjustifiably large increase in the number of fitting parameters). However, within the Supplementary Equation (4) approximation, the intensity of a satellite does not depend on ϕ, so we cannot from these diffraction data derive its value in the case that the CDW is locally commensurate

Supplementary Note 3: Visualisation of the models allowed by group theory

The YBCO unit cell is centrosymmetric about the yttrium position and has a mirror plane perpendicular to **c** at the yttrium layer between the two halves of the CuO_2 bilayer. There is also a mirror plane at the layer containing the CuO chains. For the CDW models which might fit the data, the displacement patterns must have mirror planes which coincide with one or other of the crystal mirror planes. In Supplementary Figure 3 we give a schematic representation of the symmetry of the patterns given by Group theory[8]. Models A_3 and B_3 (Supplementary Figures 2(b), 3) have basal plane displacements centred on the Y layer, and the displacements would therefore have equal amplitudes in the CuO_2 layers on either side. The next unit cell along **c** is in antiphase, which entails a zero value in the CuO chain layer of displacements of this type. However, it is clear that the basal plane compression and expansion which are opposite in adjacent cells along ***c*** must also entail **c**-axis shear displacements, which are even about the CuO chain layer. The variation along the **δ**–direction of these displacements must have a phase difference of $\pi/2$ from those in the basal plane. Models A_1 and B_1 (Supplementary Figures 2(a), 3) interchange the two crystal mirror planes, so have shear displacements along **c** in the Y/CuO_2 bilayer region, which are even about the Y layer. Basal plane displacements are odd about this plane. We emphasise that Supplementary Figure 2 represents the *symmetry* of the displacements. Since YBCO contains many different ions, the displacements of individual ions may in fact be parallel or antiparallel to the arrows in Supplementary Figure 2. The fits given in Table 1 show that this is indeed the case.

Supplementary Note 4: Determining the absolute magnitude of CDW displacements by comparison with lattice intensities

The absolute values of the displacements were estimated by comparing the satellite intensity fit with a fit of crystal Bragg peak intensities. A set of ~50 mid-intensity peaks (0.7 to 15 % of the strongest - (006)) were chosen (strong peaks may suffer from intensity errors due to extinction, and weak ones only arise from accidental cancellation of many different amplitudes). Their **Q**-space integrated intensities (normalised to fluorescence, and multiplied by the attenuation factor used) were fitted using the same *Q*-

dependent scattering amplitudes as in the CDW fit, allowing ionic positions to vary, and with overall Debye-Waller factors as defined in Supplementary Equation (9) of Supplementary Note 2. The satellite intensities were placed on the same scale by multiplying the δ-integrated intensities by the ratio of the known Q-widths to the resolution widths in the two directions not scanned. The results of this calculation give displacements $u \approx 10^{-3}$ of the lattice spacing.

It should be noted that in the calculation, one has to assume what fraction of the crystal volume is occupied by each CDW, so we discuss this in detail. The similarity of the results for the ***a*** and ***b*** directions in the present paper, and their similar temperature dependence argue for the 2-**q** scenario in which both modulations occupy the same regions. The basal-plane coherence of the CDW order is quite short, probably representing the effect of pinning. The most straightforward interpretation of a recent x-ray study[9] of the different anisotropy of the coherence lengths for the two **q**s is that the CDW is in the form of stripes (1-**q**) rather than an ideal checkerboard structure. However, we believe that the study is also consistent with 2-**q** structures with finite coherence in which both modulations co-exist locally. It appears therefore that there is not yet wide agreement on the 1-**q**/2-**q** question. Another consideration is whether the CDW structures occupy the whole volume of the crystal. It is found that the coherence lengths change with temperature and magnetic field[10, 11], but not enough to conclude that the CDWs occupy very small regions of the sample which then grow in size. Therefore, in calculating the magnitude of ionic displacements, we shall for concreteness assume that 2-**q** CDW order is present throughout the whole crystal. This assumption only affects numerical estimates of the magnitude of the displacements; all qualitative features of the CDW components are independent of this assumption. For instance, if each mode were present in domains, each only occupying half the volume, this would give a $\sqrt{2}$ larger calculated amplitude.

It is difficult to put an error on the resulting amplitudes because of the huge (and therefore uncertain) attenuation factors applied to the crystal Bragg peaks, the effects of extinction and the very different extent in **Q**-space of the crystal and CDW signals. Apart from the $\sqrt{2}$ factor above and the random errors

in the fits, this might result in an additional ~50% systematic error on the absolute scale of the displacements.

Supplementary Note 5: Establishing satellite intensities: further experimental details

The scattering plane for the four-circle diffractometer at the XMaS beamline[12] was vertical to avoid any corrections for the horizontal polarisation of the incoming beam. The sample was mounted in a closed-cycle cryostat, with its **c**-axis parallel to the 4-circle phi rotation axis and measurements were carried out in reflection from the flat **c**-face of the crystal. The detector had an energy resolution which allowed the elastic scattering to be separated completely from the Cu fluorescence from the sample at 8.1 & 8.9 keV. The omega circle was set in the 'bisecting' position (or as close as allowed by geometrical restrictions at large ℓ). This meant that the incoming and detected beams were at the same angle to the **c**-face of the crystal, which allowed normalisation by the fluorescence signal, as described below. The background counts B (which were always larger than the signal) were found to be proportional to the solid angle subtended by the detector at the sample. The signal S was also proportional to this solid angle as long as the resolution was smaller than the width of the CDW satellite. Therefore the detector slits were set to make a square detector aperture with width ~ the half-width-half-maximum of the CDW signal; this optimised the signal to noise ratio $S/\sqrt{B}$. It also meant that the **δ**-width of the satellites was dominated by the CDW correlation length, and hence showed negligible variation with **Q**. Each time measurements were made near a different (h, k, ℓ), the cryostat position was adjusted by fractions of a mm to maximise the Cu fluorescence from the sample. This ensured that the sample was centred on the incoming beam which had 0.4×0.4 mm^2 cross section. Before measuring a set of CDW satellites, the exact (h, k, ℓ) values of a lattice peak were measured (with a strongly attenuated beam) to remove any slight errors of centring of the diffractometer circles. Nearby CDW peaks were then scanned parallel to **δ**, through the positions $(h, k, \ell\pm½) \pm \delta$. The intensities of weak CDW peaks were established by fitting the scan along the **δ**-direction with a Gaussian of fixed width centred on the known δ-value of the CDW mode, with a smoothly-varying cubic polynomial background. Our careful centring allowed the width and centre of the

peak to be fixed, which enabled reliable fits of weak peaks. Fits of stronger peaks, with the same smooth backgrounds established the value of the δ-width to be used for all peaks and established that Gaussian or Lorentzian peak shapes gave equally good fits. The positions of stronger peaks were allowed to vary by up to 10% of the Gaussian (standard deviation) width if that improved the χ^2, although this made very little change in the fitted intensity. By visual examination of the fit, or examination of 150 K measurements, or by the χ^2 of the fit, spurious peaks were removed from the list of measured satellites. It was found that Poisson noise on strong backgrounds could occasionally result in fits showing an apparent intensity up to ±5% of the strongest peaks observed. To avoid distortion of the final results by the physically-impossible negative intensities, these were set no lower than -2%, and the error in all intensities less than 5% of the maximum was set at 5%; the errors on the other fitted intensities were kept at those given by the fits, multiplied by a factor of 1.25 to allow for systematic errors and to bring χ^2 close to unity. The resulting list of intensities, weighted using their errors, was fitted to our CDW models by varying the displacements $\{\boldsymbol{u}_j\}$ to minimise χ^2. Maps of all the satellite intensities and fits to them are shown in Supplementary Figure 4.

Use of Fluorescence for removal of sample illumination effects

When performing X-ray diffraction by reflection from a flat surface, the depth of sample contributing to the signal depends on the absorption coefficient μ, and the angle α between beam and surface. If the diffractometer is operated so that α has the same value for incident and diffracted beams, then the effective sample illumination depth is $\sin(\alpha)/2\mu$.The fluorescence from the sample follows the same path as the diffraction signal, but its magnitude is determined by the absorption coefficient μ for the incident beam and a different μ_f for the outgoing fluorescent X-rays. Hence, the effective illumination depth is $\sin(\alpha)/(\mu+\mu_f)$. However, if we take the ratio of these two illumination depths, we obtain $2\mu/(\mu+\mu_f)$, which is independent of angle. Hence, sample illumination effects on the CDW intensity can be removed by normalising the diffraction signal by the fluorescence counts – under our condition that the angles of incidence and exit from the surface are equal. To avoid errors arising from the small

illumination depth at grazing incidence, measurements were limited to $\alpha > 10°$, which set a lower limit on $\ell \sim 3.5$.

High energy X-ray results

Data have also been taken on this sample for a small selection of **Q**-points with high energy X-rays[13, 14]. Those results are in reasonable accord with our low energy data, and showed the distinctly different responses for the two CDW modes. However, they were not extensive enough to obtain the fits described here. We have not included the high-energy data in the fit, due to the difficulty of cross-calibration between data taken at one instrument in transmission and another in reflection.

Supplementary References

1. Abbamonte, P. *et al.* Charge modulations versus strain waves in resonant X-ray scattering. *Phys. Rev. B* **74**, 195113 (2006).

2. Johannes, M. D., and Mazin, I. I. Fermi surface nesting and the origin of charge density waves in metals. *Phys. Rev. B* **77**, 165135 (2008).

3. Casalta, H. *et al.* Neutron-scattering determination of the structural parameters versus oxygen content of $YBa_2Cu_3O_{6+x}$ single crystals *Physica* C **258**, 321-330 (1996)

4.Brown, P. J. *et. al.* in *International Tables for Crystallography*, Vol. C (ed. Wilson, A. J. C.) Ch. 6 (Kluwer Academic Publishers, 1992).

5. Amirkhanyan, Z. *et al.* Analyzing structure factor phases in pure and doped single crystals by synchrotron X-ray Renninger scanning *J. Appl. Cryst.* **47**, 160–165, (2014).

6. Cromer, D. T. and Liberman, D. Relativistic Calculation of Anomalous Scattering Factors for X Rays *J. Chem. Phys.* **53**, 1891 (1970).

7. Fujita, K. *et al.* Simultaneous Transitions in Cuprate Momentum-Space Topology and Electronic Symmetry Breaking. *Science* **344**, 612-616 (2014).

8. Campbell, B. J., Stokes, H. T., Tanner, D. E. and Hatch, D. M. ISODISPLACE: An Internet Tool for Exploring Structural Distortions. *J. Appl. Cryst.* **39**, 607-614 (2006).

9. Comin, R. *et al.* Broken translational and rotational symmetry via charge stripe order in underdoped $YBa_2Cu_3O_{6+y}$. *Science* **347**, 1335-1339 (2015).

10. Ghiringhelli, G. *et al*. Long-Range Incommensurate Charge Fluctuations in $(Y,Nd)Ba_2Cu_3O_{6+x}$. *Science* **337**, 821-825 (2012).

11. Chang, J. *et al.* Direct observation of competition between superconductivity and charge density wave order in $YBa_2Cu_3O_{6.67}$. *Nature Physics* **8**, 871-876 (2012).

12. Brown S. D. *et al.* The XMaS beamline at ESRF: instrumental developments and high resolution diffraction studies. *J. Synch. Rad.* **8**, 1172-1181 (2001); http://www.xmas.ac.uk.

13. Blackburn, E. *et al.* X-ray Diffraction Observations of a Charge-Density-Wave Order in Superconducting Ortho-II $YBa_2Cu_3O_{6.54}$ Single Crystals in Zero Magnetic Field. *Phys. Rev. Lett.* **110**, 137004 (2013).

14. Huecker, M. *et al.* Competing charge, spin, and superconducting orders in underdoped $YBaCuO_y$. *Phys. Rev. B* ***90***, 054514 (2014).
